# Epidemics of HIV, HCV and syphilis infection among synthetic drugs only users, heroin-only users and poly-drug users in Southwest China

**DOI:** 10.1038/s41598-018-25038-y

**Published:** 2018-04-26

**Authors:** Shu Su, Limin Mao, Jinxian Zhao, Liang Chen, Jun Jing, Feng Cheng, Lei Zhang

**Affiliations:** 1 School of Public Health and Preventive Medicine, Faculty of Medicine, Nursing and Health Sciences, Monash University, Melbourne, VIC Australia; 2 Center for Social Research in Health, Arts and Social Sciences, UNSW Australia, Sydney, NSW Australia; 3 Division of HIV/AIDS and STI Control, Centers for Disease Control and Prevention, Yuxi Prefecture, Yunnan China; 4 Research Center for Public Health, School of Medicine, Tsinghua University, Beijing, China; 5 Melbourne Sexual Health Centre, Alfred Health, Melbourne, Victoria Australia

## Abstract

The number of poly-drug users who mix use heroin and synthetic drugs (SD) is increasing worldwide. The objective of this study is to measure the risk factors for being infected with hepatitis C (HCV), human immunodeficiency virus (HIV) and syphilis among SD-only users, heroin-only users and poly-drug users. A cross-sectional study was conducted in 2015 from a national HIV surveillance site in Southwest China, 447 poly-drug, 526 SD-only and 318 heroin-only users were recruited. Poly-drug users have higher drug-use frequency, higher rates of drug-sharing and unsafe sexual acts than other users (*p* < 0.05). About a third (36.7%) of poly-drug users experienced sexual arousal due to drug effects, which is higher than the rate among other drug users. Poly-drug users had the highest prevalence of HIV (10.5%) and syphilis (3.6%), but heroin-only users had the highest prevalence of HCV (66.0%) (all *p* < 0.05) among three groups. Logistic regression shows among poly-drug users, having sex following drug consumption and using drugs ≥1/day were the major risk factors for both HIV (Adjusted odds ratio (AOR) = 2.4, 95% CI [1.8–3.4]; 2.3, [1.6–3.1]) and syphilis infection (AOR = 4.1, [2.1–6.9]; 3.9, [1.8–5.4]). Elevated risk of both HIV and syphilis infection have been established among poly-drug users.

## Introduction

In 2016, an estimated 250 million people worldwide used non-prescribed drugs and about 29.5 million of those drug users suffered from drug use disorders^1^. While the number of drug users has continued to grow over the past five years, but access to health services underpinned by harm reduction principles remained low (about one in six persons with drug use disorders have such access)^1^. Human immunodeficiency virus (HIV) and hepatitis C (HCV) infection is a major concern among injection drug users (IDU), as injection drug use carries a high risk of blood-borne transmission of infections, it is reported that HIV infection risk per needle sharing acts can reach 2.38%^2^. Consequently, specific disease burdens in this population are disproportionally high: in 2015, approximately 13.3% of people who inject drugs were living with human immunodeficiency virus (HIV), and 50.8% were living with chronic hepatitis C (HCV) infection^1^.

The widespread use of synthetic drugs (SDs) has also caused rising concern globally. Synthetic drugs are processed, artificial chemicals that mimic the effects of traditional recreational drugs. They include both amphetamine-type stimulants (e.g., methamphetamine, ketamine, ecstasy) and new psychoactive substances (drugs with chemical structures similar to those of known illegal synthetic drugs that produce similar effects to illegal drugs)^3–6^. Consumption of these drugs has increased rapidly since 2009, first in North America and Europe and gradually spreading to the rest of the world^7^. In 2015, problematic use of SDs was reported in 95 countries, with the largest proportion seen in East and South-East Asian countries^1^. Further, studies in well-known drug trafficking zones in the Asia-Pacific region, including Thailand, Myanmar and Malaysia, have shown an alarming increase in SD consumption, with a particularly notable increase among young people^8–11^.

Despite its strong prohibitive drug policies, China, has also conformed the shifting global drug use patterns^12^. Recreational drugs in China consisted primarily of traditional opiates, SD, and toxic medicinal drugs, with the former two types accounting for more than 90% of the drug market. By 2016, 2.5 million drug users were registered with the Chinese authority (i.e., those who are arrested by the police or who voluntarily register with community and detoxification centre, and the number is updated and published each year by China National Narcotics Control Commission and only includes alive and current drug users), which represents a significant increase from the 1.1 million drug users registered in 2008^13^. Among all drug users, there has been an unprecedented increase in the proportion of people using SD: from 20% in 2008 to 61.3% in 2016 (i.e., the proportion of SD use has tripled over a nine-year period and now accounts for more than half of all drug use; SD use has become dominant instead of heroin use)^13,14^. Due to the substantial stimulatory and euphoric effects of SDs, they are commonly used in nightclubs and among sexually active populations for recreational purposes^15,16^. Drug users are more likely to engage in unprotected sex, and with more and more drug-facilitated sexual assaults are occurring under the influence of mind-altering SDs^17,18^. It has been estimated that the transmission risk of HIV, a known sexually transmitted infection (STI), per unprotected sexual acts is 0.3%^19^. So the rising popularity of SDs may fuel HIV and STI epidemics, especially syphilis, which has become increasingly prevalent in China during the past decade.

It has been documented that 30–40% of heroin users in China also use SDs, and that proportion is expected to grow rapidly in the coming years^20,21^. In this study, we defined poly-drug use as simultaneous mixed use of an SD and heroin. In contrast with SD-only users, poly-drug use is associated with more harmful drug effects and high-risk behaviours^22^. Because drugs have acute and chronic effects on bodily functioning, the combined use of multiple drugs can magnify their pharmacological effects^23^, for example, the co-use of cocaine and heroin has a synergistic impact on reducing norepinephrine release and reuptake^24^. So the same dosage of poly-drugs can cause stronger stimulation than either heroin or SD alone, resulting in users having a higher possibility of engaging in high-risk behaviours, such as condomless sex and/or group sex^22,25^. Moreover, in China, poly-drug users are more likely to inject drugs and share injection equipment than SD-only users. This behaviour also leads to higher risk of blood-borne viruses.

In order to curb the rapid increase of BBVSTIs transmission nationally, the Chinese government announced in 2009 a 10-year syphilis plan (2010–2020) to control syphilis incidence and more recently introduced a 5-year HIV plan (2017–2022) to reduce HIV infections from the sexual and drug injection transmission routes. The World Health Organization has set an ambitious goal to eliminate HCV by 2030^26^. However, in contrast to these national and international efforts to decreasing the disease burden of blood-borne viruses and sexually transmitted infections (BBVSTIs) disease burden, the rapid increase of SD and poly-drug use in recent years is further fueling these epidemics. In fact, an alarmingly high prevalence of HIV (6.0%) and syphilis (7.8%) infection has already been observed among drug users^27–29^, and meta-analysis estimated an HCV prevalence of 67.0% among injection drug users and 60.1% among all drug users in China^29^. Because the expanding drug-using population can potentially transmit BBVSTIs to the general population, a better understanding of the risk factors associated with HIV, HCV and syphilis infection, and the adverse effects associated with different kinds of drug users, are urgently needed to develop effective harm reduction strategies to slow further BBVSTIs transmission in a rapidly changing drug era^20,30^.

Yunnan province is a large province in Southwest China and borders the ‘Golden Triangle’, which is a major heroin manufacturing site at the borders of Myanmar, Thailand, Laos and Vietnam^31^. Yunnan province accounts for 3.3% of the total population in China and due to the ease of access to a wide range of new and often affordable recreational drugs across its border, it had 160,000 registered drug users in 2014 (approximately 5% of all registered drug users in China)^32^. According to China’s 2017Annual drug report on drug control, the top five drugs abused in China are methamphetamine, heroin, magu (a mixture of methamphetamine and caffeine)^33^, ketamine and methadone^34^. These drugs are all commonly and popularly used in Yunnan province, so the results of this study may be extrapolated to drug users in other parts of China. We consider this area an optimal study setting to assess the changing patterns of drug use and the impacts of those patterns on local HIV, HCV, and syphilis epidemics. This study aims to assess the prevalence of HIV, HCV and syphilis infection and their associated risk factors among poly-drug users, SD-only users and heroin-only users and compare the differences in socio-demographic characteristics, drug use practices and drug-related effects among these three groups.

## Results

### Socio-demographic characteristics

A total of 1, 291 new entrants at the largest detoxification centre in Yunnan Province were enrolled in the study, 447 (34.6%) were poly-drug users, 526 (40.7%) were SD-only users, and 318 (24.6%) were heroin-only users according to their self-reported drug consumption patterns. Education status is similar in three groups, however, gender, age, marriage status, and monthly income demonstrated a graded distribution among three groups, poly-drug users were consistently ranked between SD-only users and heroin-only users. As shown in Table 1, the proportion of female drug users was highest among the SD-only users (114, 21.7%) and lowest among the heroin-only users (27, 8.5%) (p < 0.05). SD-only users were the youngest (median age of 24 years, interquartile range(IQR) 21–27 years old), had the highest proportion earning at least 2,000 RMB [equivalent to 300 USD] monthly (42.8%) and had the highest unmarried rate (83.7%); in contrast, heroin-only users were the oldest (29, IQR 25–35), with the highest married rate (26.4%) and the highest percentage of people with a monthly income lower than 2000 RMB (78.3%) among three groups (Table 1).

**Table 1:** Baseline demographic characteristics, drug use pattern and infection status, stratified by poly-drug use, synthetic drug only and heroin-only users.

Items	Total	Poly-drug (%)	Synthetic drug only (%)	Heroin only (%)	P-value	X^2^
***Demographic characteristics***						
**Gender (%)**					<0.01*	25.63
Female	210 (16.3)	69 (15.4)	114 (21.7)	27 (8.5)		
Male	1081 (83.7)	378 (75.5)	412 (78.3)	291 (91.5)		
**Age (IQR)**	25 (21–29)	26 (22–32)	24 (21–27)	29 (25–35)	0.03*	4.16
**Education (%)**					0.51	1.32
Senior high and above	151 (11.7)	46 (10.3)	66 (12.5)	39 (12.3)		
Junior high and below	1140 (88.3)	401 (89.7)	460 (87.5)	279 (87.7)		
**Marriage status (%)**					<0.01*	48.12
Single	970 (75.1)	331 (74.0)	440 (83.7)	199 (62.6)		
Married	228 (17.7)	80 (17.9)	64 (12.2)	84 (26.4)		
Widowed	93 (7.2)	36 (8.1)	22 (4.2)	35 (11.0)		
**Monthly income (RMB)**					<0.01*	43.18
<2000	864 (66.9)	314 (70.2)	301 (57.2)	249 (78.3)		
>=2000	427 (33.1)	133 (29.8)	225 (42.8)	69 (11.7)		
***Drug used behaviours***						
**Age for initiating drug use (years)**	20 (17–25)	20 (17–25)	20 (17–24)	19 (18–25)	0.31	1.11
**Duration of drug use (years)**	3 (1–6)	4 (3–6)	2 (1–4)	6 (3–8)	<0.01*	26.23
**Ever injected drugs in last three months (%)**					<0.01*	280.35
Yes	610 (47.2)	277 (62.0)	38 (7.2)	295 (92.8)		
No	681 (52.8)	170 (38.0)	488 (92.8)	23 (7.2)		
**Type of drug used in the last three months (%)**					<0.01*	217.43
Ecstasy	123 (9.5)	82 (18.3)	41 (7.8)	0 (0)		
Methamphetamine	208 (16.1)	139 (31.1)	69 (13.1)	0 (0)		
Ketamine	115 (8.9)	91 (20.4)	24 (4.6)	0 (0)		
Magu	870 (67.4)	381 (85.2)	489 (93.0)	0 (0)		
Heroin	765 (59.3)	447 (100)	0 (0)	318 (100)		
**Reasons for initiating drug use (%)**					<0.01*	8.13
Mental pleasure	673 (52.1)	267 (59.7)	195 (37.1)	211 (66.4)		
Perceiving no addiction	200 (15.5)	46 (10.3)	125 (23.8)	29 (9.1)		
Enhancing sex function	181 (14.0)	79 (17.7)	76 (14.4)	26 (8.2)		
Curiosity	105 (8.1)	13 (2.9)	69 (13.1)	23 (7.2)		
Fashion	132 (10.2)	42 (9.4)	61 (11.6)	29 (9.1)		
**Frequency of drug use in the last three months (%)**					<0.01*	101.20
More than once a day	133(10.3)	64 (14.3)	24 (4.6)	55 (14.2)		
Once a day	184 (14.3)	98 (21.9)	44 (8.4)	42 (13.2)		
Once or more than once a week	758 (58.7)	251 (56.2)	308 (58.6)	189 (62.6)		
Once or more than once a month	216 (16.7)	34 (7.6)	150 (28.5)	32 (10.1)		
**Number of persons shared drugs in the last act(mean)**	2.4 ± 0.7	2.9 ± 1.1	2 ± 0.8	1.7 ± 0.5	0.01*	7.26
**With whom shared drugs (%)**					0.01*	8.19
Alone	162 (12.5)	44 (9.8)	41 (7.8)	77 (24.2)		
Sexual partner	186 (14.4)	81 (18.1)	71 (13.5)	34 (10.7)		
Friends	943 (73.0)	322 (72.0)	414 (78.7)	207 (65.1)		
***Infection Status***						
**HIV (%)**					<0.01*	8.33
Positive	80 (6.2)	47 (10.5)	6 (1.1)	27 (8.5)		
Negative	1211 (93.8)	400 (89.5)	520 (98.9)	291 (91.5)		
**Syphilis (%)**					0.02*	3.52
Positive	28 (1.9)	16 (3.6)	8 (1.5)	4 (1.3)		
Negative	1263 (98.1)	431 (96.4)	518 (98.5)	314 (98.7)		
**HCV (%)**					<0.01*	15.22
Positive	483 (37.4)	249 (55.7)	24 (4.6)	210 (66.0)		
Negative	808 (62.6)	198 (44.3)	502 (95.4)	108 (34.0)		

### Drug-related practices

A striking difference related to drug injection was observed among the three groups: while 62.0% (277) of poly-drug users reported ever injecting any drugs, almost all heroin-only users (92.8%, 295), but only 7.2% (38) of SD-only users reported a history of drug injection (*p* < 0.01). Poly-drug users reported higher drug use frequency, with 36.2% of them using at least once-daily over the last three months compared with SD-only users (13.0%) and heroin-only users (27.4%). Poly-drug users exhibited the highest rate of drug sharing, the average number of poly-drug users shared drugs was 2.9 ± 1.1 in the last drug use, while SD-only and heroin-only users shared drug among 2 ± 0.8 and 1.7 ± 0.5 people in the last act, respectively. The age of first drug use was similar among the three groups, but heroin-only users had a longer duration of drug use (median 6 years, IQR 3–8 years) than the other drug users. Magu (67.4%) was by far the most commonly reported drug of choice in the past three months, followed by heroin (59.3%), methamphetamine (16.1%), ecstasy (9.5%) and ketamine (8.9%).

### Adverse reactions and sexual activities following recent drug consumption

As shown in Table 2, poly-drug users reported a higher rate (36.7%, 164) of sexual arousal effects following drug use than SD-only users (19.4%, 102) and heroin-only users (21.7%, 69) (*p* < 0.01). Corresponding to the sexual arousal rate, the number of users engaging in sexual activities following drug use in the past month was significantly higher among poly-drug users (251, 56.2%) than SD-only users (207, 39.4%) and heroin-only users (96, 30.2%) (*p* < 0.01). Additionally, sex with a non-regular partner (i.e., one-night-stands) after recent drug consumption in the past month was highest among poly-drug users engaging in sexual activity following drug use (54.6%, 137, *p* = 0.02). Of interest, reported rates of engaging in condomless sex did not differ significantly between the three groups. Hallucinations were more likely to be reported by SD-only users (37.1% vs. 27.1% in poly-drug users and 19.8% in heroin-only users, *p* < 0.05). Violent acts, however, were more likely to be committed by heroin-only users (31.4%, 100) (*p* < 0.01) (Table 2).

**Table 2:** Drug effects and sexual behaviours immediately after drug use.

Items	Total	Poly-drug (%)	Synthetic drug only (%)	Heroin only (%)	P-value	X^2^
***Drug reactions***						
Sexual arousal	335 (25.9)	164 (36.7)	102 (19.4)	69 (21.7)	<0.01*	41.59
Uncontrolled body movements	189 (14.6)	98 (21.9)	148 (28.1)	21 (6.6)	<0.01*	130.08
Constant hyperaction	194 (15.0)	34 (7.6)	85 (16.2)	75 (23.6)	<0.01*	38.05
Hallucination	379 (29.4)	121 (27.1)	195 (37.1)	63 (19.8)	<0.01*	30.20
Violence	230 (17.8)	77 (17.2)	53 (10.1)	100 (31.4)	<0.01*	61.98
Insomnia	797 (61.7)	306 (68.5)	351 (66.7)	140 (44.0)	<0.01*	56.32
***Sexual behaviours***						
**Had sex immediately after drug use**					<0.01*	55.72
Yes	554 (42.9)	251 (56.2)	207 (39.4)	96 (30.2)		
No	737 (57.1)	196 (43.8)	319 (60.6)	222 (69.8)		
**Type of sexual partner (among those who had sex)**					0.02*	8.39
Regular	287 (51.8)	114 (45.4)	122 (58.9)	51 (53.1)		
Casual	267 (48.2)	137 (54.6)	85 (41.1)	45 (46.9)		
**Condom use (among those who had sex)**					0.5	1.37
Yes	159 (28.7)	74 (29.5)	54 (26.1)	31 (32.3)		
No	395 (71.3)	177 (70.5)	153 (73.9)	65 (67.7)		

### Prevalence of confirmed HIV, HCV and syphilis infections

As shown in the last section of Table 1, poly-drug users had the highest prevalence of HIV (10.5%, 47), followed by heroin-only users (8.5%, 27) and SD-only users (1.1%, 6, *p* < 0.01). More than half of poly-drug users were confirmed (either tested at the time of the survey or verified through health records) to be HCV-positive (55.7%, 249). The highest HCV prevalence was observed among heroin-only users (66.0%, 210) compared with no more than 5% of SD-only users (4.6%, 24, *p* < 0.01). Similarly, but on a much smaller scale, confirmed rates of syphilis infection (3.6%, 16) were highest in the poly-drug users compared to SD-only users (1.5%, 8) and heroin-only users (1.3%, 4, *p* = 0.02) (Table 1).

### Factors associated with confirmed HIV, HCV and syphilis infections

**Figure 1:**
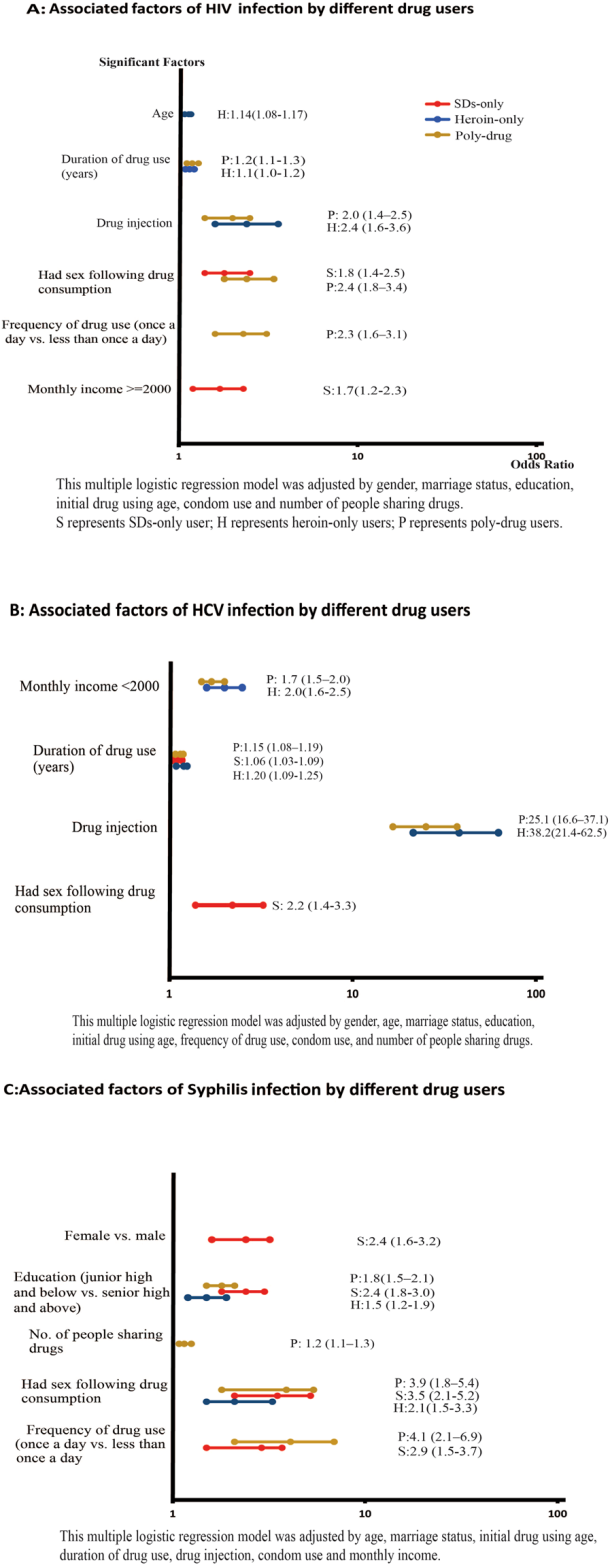
Associated factors of HIV, HCV and syphilis infection by different drug users.

Engaging in sex following drug consumption and using drugs ≥1/day were the main risk factors for HIV infections (adjusted odds ratio (AOR) = 2.4, 1.8–3.4 and AOR = 2.3, 1.6–3.1, respectively) among poly-drug users. Longer duration of drug use (AOR = 1.15, 1.05–1.26) and injecting drugs (AOR = 2.0, 1.4–2.5) were independently associated with confirmed HIV infection among poly-drug users. For the SD-only users, engaging in sex following drug consumption (AOR = 1.8, 1.4–2.5) and having a monthly income higher than 2000 RMB (AOR = 1.7, 1.2–2.3) were both positively correlated with HIV infection. Heroin-only users with elder age (AOR = 1.14, 1.08–1.17), longer drug use duration (AOR = 1.07, 1.04–1.19) and those who inject (AOR = 2.4, 1.6–3.6) had a higher risk of HIV infection (Fig. 1A). This model was adjusted by gender, marriage status, education, initial drug using age, condom use and the number of people sharing drugs (Supplementary file, dataset 1).

Injecting drugs remained the strongest risk factor associated with HCV infection among poly-drug users (AOR = 25.1, 16.6–37.1). In addition, poly-drug users with monthly income lower than 2000 RMB (AOR = 1.7, 1.5–2.0) and longer duration of drug use (AOR = 1.15, 1.08–1.19) had a higher risk of being HCV infected. Notably, engaging in sex following drug consumption (AOR = 2.2, 1.4–3.3) and duration of drug use (AOR = 1.06, 1.03–1.09) were two factors associated with HCV co-infection among SD-only users. Similarly to poly-drug users, injecting was a high-risk factor contributing to HCV infection among heroin-only users (AOR = 38.2, 21.4–62.5). Other independent variables correlated with HCV-infection among heroin-only users included lower monthly income (AOR = 2.0, 1.6–2.5) and a longer duration of drug use (AOR = 1.20, 1.09–1.25) (Fig. 1B). The model was adjusted by gender, age, marriage status, education, initial drug using age, the frequency of drug use and condom use (Supplementary file, dataset 1).

Consistent with the above results for HIV infection, engaging in sex following drug consumption and using drugs once or more per day (AOR = 3.9, 1.8–5.4 and AOR = 4.1, 2.1–6.9, respectively) were the main risk factors associated with syphilis infections among poly-drug users. Among this population, lower education (AOR = 1.8, 1.5–2.1) and drug-sharing rate (AOR = 1.15, 1.08–1.25) were also associated with syphilis infection. For SD-only users, female drug users (AOR = 2.4, 1.6–3.2), with lower education (AOR = 2.4, 1.8–3.0), who engaged in sex following recent drug consumption (AOR = 3.5, 2.1–5.2), and used drugs more than once daily (AOR = 2.9, 1.5–3.7) were more likely to be infected with syphilis. For heroin-only users, lower education (AOR = 1.5, 1.2–1.9) and engaging in sex after drug consumption (AOR = 2.1, 1.5–3.3) were positively correlated with syphilis infection (Fig. 1C). The model was adjusted by age, marriage status, initial drug using age, duration of drug use, drug injection, condom use and monthly income (Supplementary file, dataset 1).

## Discussion

In this study, poly-drug users demonstrated the highest prevalence of HIV and syphilis infections, while heroin-only users demonstrated the highest prevalence of HCV infection among the three groups. Engaging in sex following drug consumption and using drugs once or more per day appear to be the major risk factors for both HIV and syphilis infection among poly-drug users. Drug injection remains the main risk factor contributing to HCV infections among heroin-only users and poly-drug users. Notably, unsafe sexual behaviour is the major factor contributing to increased risk of HIV, HCV and syphilis infection among all SD users. Elevated risks of both HIV and syphilis infection have been established among poly-drug users. Harm reduction interventions should target the growing population of poly-drug users for effective control of BBVSTIs epidemics.

Our findings indicate that poly-drug use may result in high-risk behaviours and is becoming the leading cause of BBVSTI transmission. First, poly-drug users have the highest drug-use frequency among the three groups of drug users. This may be due to using of multiple drugs is to reduce unwanted side-effects or withdrawal symptoms, so poly-drug users may have to use drugs more frequently^35^. Another reason may be that the stimulant effects of synthetic drugs can mask the signs of heroin overdose (e.g. respiratory depression), leading users to mistakenly feel that they can take more to maximize satisfaction^36^. Furthermore, our study shows that poly-drug users are more likely to share drugs than other sole drug users. This is perhaps because some drug users especially young people initiate poly-drug use in social situation for instance at parties where multiple drugs are used due to peer pressure^35^, so they may share drugs together among their circle of friends. This may lead to higher risk among poly-drug users of using contaminated syringes, engaging in unprotected sex and increasing the drug use frequency than the heroin-only and SD-only users. Notably, the comparison of demographic characteristics showed that SD-only users are the youngest and have the shortest history of exposure to drug consumption among all the drug users considered in the study. This may be partly explained by the fact that SDs have only become widely available in China in recent years^37^. Previous studies in other countries have reported that SD users are likely to become poly-drug users^38,39^, so further studies could be conducted to confirm whether drug use duration is linearly related to the possibility that SD-only users will become poly-drug users.

Compared with heroin-only and SD-only users, poly-drug users were more likely to experience sexual arousal following drug consumption. Correspondingly, poly-drug users reported a higher incidence of subsequent sexual behaviours and sex with casual partners. However, the prevalence of condomless sex in such contexts was not significantly different among the three groups and all exhibited rates higher than 60%. This situation raises a severe concern with regard to transmitting BBVSTIs as using drugs to enhance sex becomes more prevalent among drug users; in addition, it has been found that drug use can result in the lowering of inhibitions among those who would normally use condoms,leading to unprotected sex^40^. Our results suggest that unsafe sexual behaviours may contribute more to HIV infection than injecting behaviours among poly-drug users, which could indicate that unprotected sexual acts have become the dominant transmission route in this population. Furthermore, poly-drug users have higher likelihood of injecting SDs than SD-only users, in a process called ‘slamsex’, which means injecting rather than snorting or orally taking SDs in order to get a more intense high from synthetic drugs^41^. This behaviour may lead to a more intense high and thus to more serious behavioural consequences. In addition, needle sharing for ‘slamsex’ can also spread BBVSTIs^41^. Effective interventions should be implemented for poly-drug users who face this dual risk.

In the present study, hallucinations and uncontrolled body movements were more commonly reported as adverse reactions after drug consumption by SD-only users than by their counterparts. This is also the main symptom observed among poly-drug users, a result that is consistent with the documented side effects of SDs, which can cause either short-term delirium or long-term psychotic reactions^42–44^. However, with the increasing number of SD classes on the market, the potential combinations of different drugs may lead to more side effects and even lethal outcomes among poly-drug users. Violent acts are also a major concern associated with heroin-only users, as uncontrolled violent behaviours are often related to sexual violence^45,46^, which is a risk factor associated with sex crimes and STI transmission^47,48^. This drug influence was also observed among poly-drug users.

Given the rapid increase in synthetic drug and poly-drug use and its serious consequences, interventions to mitigate harm are imperative. First, current synthetic drug users should be encouraged to cease SD use. However, quitting synthetic drug use is difficult as withdrawal has been shown to cause psychological rather than physical symptoms^49^. Globally, it is common to help drug users by using psychological approaches including motivational interviewing, meditation, family support and light physical activities such as yoga^50^. It is necessary to implement national intervention programs targeting SD users in China. For example, China has developed a national methadone maintenance treatment program for people with opioid dependence.

Second, drug policies should be altered to protect and treat drug users. Currently, in line with similar policies aimed at controlling heroin use, China has announced strict policies to reduce use of synthetic drugs. Chinese drug law states that those who smuggle, sell, transport, or manufacture methamphetamine or other narcotics in an amount greater than fifty grams can be sentenced to fifteen years in prison, life imprisonment, or death^51^. However, in light of the ever-increasing number of drug users and the amounts of drugs seized by police in recent years, the Chinese government should also consider a new experimental trial using more compassionate and humanitarian methods of SD harm reduction. As an example, Portugal decriminalised the acquisition, possession, and use of small quantities (up to a 10-day supply) of all psychoactive drugs for personal use in 2001^52^. The general view is that the strategy has caused a reduction in the rates of drug use, drug-related deaths and blood-borne viruses among existing users^53^. In 2009, the government of Mexico also enacted a law to decriminalise the possession of small amounts of drugs, which has had positive effects on the control of drug use^54^. Although such drug-policy reform remains controversial, its results merit conducting a pilot study that would combine the decriminalisation policy with intervention to scale up BBVSTIs testing, treatment and prevention programs for SD users as an alternative to harsh penalties in China. This might ameliorate the negative effects of increased drug use on BBVSTIs.

Finally, promoting needle and syringe programs among drug users should be considered to reduce transmission of blood-borne diseases, as this approach has proven to be an effective intervention in other countries^55^. This study indicates that intravenous drug injection is still the primary route of HCV transmission^56,57^ for both poly-drug users and heroin-only users. Also, our research shows that drug users with lower income are more likely to be HCV-positive. This is an alarming signal, suggesting that low-income users cannot afford clean needles. Furthermore, the rate of drug sharing was observed to be higher among poly-drug users, and longer drug use duration was shown to be one risk factor associated with HCV infection^58^. It is reasonable to estimate that there will be an increasing trend of HCV infection among poly-drug users, highlighting the urgent need for clean syringes. Our study further suggests that an elevated HCV risk is attributable to sexual transmission in the context of drug use among SD-only users^59^. This is a plausible conjecture, as a recent meta-analysis showed that sexual transmission of HCV is highly probable in HIV-positive men who have sex with men^60^. Reducing high-risk sexual behaviours under drug influence may also benefit the prevention of HCV infection.

There are several limitations in this study. First, we cannot assess the specific poly-drug use of each user; for example, we could not determine whether a drug user used heroin first then used SDs or SDs first and then heroin. The order may have an impact on the prevalence of BBVSTIs. Second, practices related to recreational drug use, which is illegal in China, relied exclusively on self-reports, which can lead to recall and social desirability biases. Third, detailed reasons for consuming SDs and heroin were not collected, limiting our ability to provide further contextual information to illuminate the main findings. Despite these limitations, our study is the first in China to reveal that (a) poly-drug users have elevated risks of both HIV and syphilis infection and (b) a potential outbreak of BBVSTIs is highly likely among younger drug users who have only recently initiated SD use if the status quo continues.

## Conclusion

Our findings underscore the necessity of implementing effective harm reduction interventions and BBVSTI testing, treatment and prevention to support the chronic, complex and ever-changing needs of poly-drug users and address the potential shift to younger, more recently initiated SD-only users. Against the background of already harsh drug regulation and drug user crackdown policies for synthetic drugs and heroin in China, it has become more important to expand practical and effective harm-reduction programs for current poly-drug and SD users in order to curb the rise in BBVSTIs resulting from risky sexual behaviours and the chronic drug injection.

## Methods

### Study setting and sampling

A cross-sectional survey, as a part of the routine sentinel drug user surveillance carried out in the largest detoxification centre in Yuxi, which is also one of the Chinese national HIV sentential surveillance sites that routinely collect data from designated priority populations. China has adopted a compulsory ‘detoxification’ policy, which means that drug users arrested by the police or reported by the community are compelled to enter a detoxification center for treatment. China has 700 detoxification centers, and these centers are housing more than 350,000 drug users^61^, so the population of detoxification centers can represent the general population of drug users in China.

Data was collected on the first day of admission to the detoxification centre between mid-January and mid-December of 2015. Eligibility criteria included the following: 1) use of recreational drugs in the previous week; 2) being 18 years of age or older; 3) residing in Yuxi in the previous three months; and 4) provision of written informed consent. In this study, SD includes methamphetamine, ecstasy, ketamine, and magu. We distinguished ‘poly-drug users’ (those who used both heroin and any SD concurrently in the previous three months) from heroin-only users (never used SD before) and SD-only users (never used heroin before). We excluded drug users who used heroin and SD concurrently earlier than the previous three months to avoid bias. The participants were classified into the aforementioned three categories. Participants completed a paper survey via self-interviewing, with 15 minutes average completion time. This was followed by serological tests for HIV, HCV and syphilis confirmation and a urine test. The results of urine detection of drug residuals were used to corroborate self-reported drug consumption in the previous week.

### Ethics statement

The study reports findings from the secondary data analysis (stripped of any personal identifier) of these local routinely collected bio-behavioural surveillance data, which was approved by the Monash University Human Research Ethics Committee (CF16/942 - 2016000495). The collected data were analyzed only for the purposes of this study. No further informed consent about this study was required.

### Self-completed questionnaire, laboratory screening, and data linkage

The survey consisted of four sections: socio-demographic characteristics, patterns of recreational drug use, specific reactions following recreational drug use, and subsequent sexual behavioural patterns after drug consumption. The questionnaire was pilot-tested before the formal roll-out. Serological detection of HIV, HCV and syphilis followed standard national diagnostic guidelines in China: HIV-positivity was confirmed by Enzyme-linked immunosorbent assay (ELISA) screening and Western Blot (WB) validation^62^; HCV-positivity was confirmed by the detection of HCV antibodies through repeated, independent ELISA testing^63^; and syphilis-positivity was established by Rapid Plasma Reagin (RPR) Circle Card Test screening and Treponema Pallidum Particle Agglutination Assay (TPPA) validation. Laboratory results (performed as part of routine surveillance at community sites) and additional serological tests were then linked through a unique study ID for each participant by local CDC data experts. Those with laboratory-confirmed HIV, HCV or syphilis infections were then notified and referred to treatment whenever possible, following standard clinical referral pathways.

### Statistical analysis

For this paper, the analysis was performed on a selected sub-group of surveillance participants according to their drug use. All data were managed and analysed using the statistical software SAS 9.4 (Statistical Analysis System). Descriptive and inferential statistical analyses were performed. Medians and interquartile ranges (IQRs) were calculated to summarise numerical variables, whereas frequencies and percentages were presented to describe categorical variables. A chi-square test was built to assess key factors differentiating SD-only users, heroin-only users and poly-drug users. Three multivariable logistic regressions were used to identify factors associated with increased prevalence rates of BBVSTIs within each group. The final model was adjusted for demographic characteristics confounding, which are the variables with a p-value < 0.2 in the univariate analysis were included in the multivariate analyses. Collinearity was checked by evaluating the impact factors of all the indicator variables in multivariate analyses. A *p*-value of less than 0.05 was considered significant in the final logistic regression models.

## Electronic supplementary material

Dataset 1
